# Dynamic computed tomography angiography for noninvasive diagnosis of bow Hunter's syndrome: a case report

**DOI:** 10.31744/einstein_journal/2024RC0582

**Published:** 2024-06-13

**Authors:** Giovanna Sawaya Torre, Bruna Bonaventura Failla, Felipe Soares Oliveira Portela, Marcelo Passos Teivelis, Nelson Wolosker, Rogerio Iquizli, Carlos Augusto Ventura Pinto, Marcelo Assis Rocha, Adriano Tachibana

**Affiliations:** 1 Hospital Israelita Albert Einstein São Paulo SP Brazil Hospital Israelita Albert Einstein, São Paulo, SP, Brazil.

**Keywords:** Vertebral artery, Vertebrobasilar insufficiency, Tomography, X-ray computed, Angiography, digital subtraction

## Abstract

The focus of this case report is to technically describe a noninvasive diagnostic evaluation of bow Hunter's syndrome using a dynamic computed tomography angiography protocol and discuss its advantages. In addition, we aimed to exemplify the quality of the study by presenting images of a 3D-printed model generated to help plan the surgical treatment for the patient. The dynamic computed tomography angiography protocol consisted of a first image acquisition with the patient in the anatomic position of the head and neck. This was followed by a second acquisition with the head and neck rotated to the side that triggered the symptoms, with technical parameters similar to the first acquisition. The acquired images were used to print a 3D model to better depict the findings for the surgical team. The dynamic computed tomography angiography protocol developed in this study helped visualize the vertebrobasilar arterial anatomy, detect vertebral artery stenosis produced by head and neck rotation, depict the structure responsible for artery stenosis (*e.g*., bony structure or membranes), and study possible complications of the disease (*e.g*., posterior cerebral circulation infarction). Additionally, the 3D-printed model better illustrated the findings of stenosis, aiding in surgical planning. In conclusion, dynamic computed tomography angiography for the evaluation of bow Hunter's syndrome is a feasible noninvasive technique that can be used as an alternative to traditional diagnostic methods.

## INTRODUCTION

Bow Hunter's syndrome (BHS), also referred to as bow hunter's stroke or rotational vertebral artery (VA) occlusion syndrome, involves symptomatic vertebrobasilar insufficiency caused by mechanical occlusion or compression of the VA at the atlantoaxial or subaxial level during neck and head rotation.^(1)^

In many individuals, the VAs are not completely symmetrical in terms of size or course. This means that one VA may be larger or may take a slightly different path than the other. In approximately 50% of the population, the left VA is dominant,^(2)^ and contributes more significantly to the formation of the basilar artery; therefore, it is a relevant anatomical variation to be described in imaging studies, as it can further impact the change in blood flow when BHS occurs.

However, given the relative rarity of this syndrome, its prevalence and incidence are not well known. It frequently involves syncopal or presyncopal attacks, vertigo, and/or impaired vision, although the symptoms may vary from transient ischemic attacks to potentially devastating posterior circulation strokes, which can be repeatedly evoked by the rotation or extension of the head and neck.^(1)^

Treatment mainly depends on the severity of patient symptoms, the structure related to artery dynamic stenosis (most commonly the cervical spine bone, ligament, or membrane), and the involved VA segment (V1–V4). Treatment might be conservative (cervical rotation containment), surgical (decompression, fusion, or a combination), or more recently, endovascular intervention.^(3,4)^

Digital subtraction angiography (DSA) is the method of choice for the diagnosis of BHS, as it can show VA stenosis during head and neck rotation. However, this procedure is invasive and has limited potential for depicting adjacent vascular structures.^(5)^ Concerning alternative noninvasive methods of BHS diagnosis and evaluation, Doppler sonography has the ability to examine VAs with head and neck rotation and might assess flow changes suggestive of significant stenosis; however, it lacks the accuracy to evaluate adjacent arterial structures and central nervous system disease complications. Conventional computed tomography angiography (CTA) and magnetic resonance angiography (MRA) have optimal accuracy in depicting the head and neck, intracranial vascular anatomy (including VA dominance, an important finding in BHS), and disease. However, since these procedures are performed in an anatomical position and BHS syndrome is triggered by head and neck rotation, BHS evaluation using these techniques is suboptimal. Dynamic CTA and MRA consist of imaging a patient suspected of having BHS in the neutral position and in the position that reproduces symptoms. These techniques incorporate all the benefits of conventional CTA/MRA concerning head and neck and intracranial vascular disease evaluation, adding the possibility of detecting VA stenosis produced by head and neck rotation, as well as that of observing the structure responsible for artery stenosis (*e.g.* bony structure or membranes). Moreover, it may aid in assessing possible complications of the disease (*e.g.* posterior cerebral circulation infarction).^(6)^

Recently, Shi et al.^(7)^ highlighted the importance and implications of dynamic CTA of the head and neck in the diagnosis of BHS. However, to the best of our knowledge, a detailed technical description of the acquisition protocol for assessing BHS is lacking.

Therefore, this study aimed to provide a detailed description of the dynamic CTA acquisition protocol for BHS investigation, discuss its benefits, and illustrate the quality of the study using images of a 3D-printed model required by the surgical team.

## METHODS

Dynamic CTA for BHS evaluation was performed using a protocol directed toward the analysis of the arterial vessels of the neck. For contrast injection, an iodinated, nonionic, low osmolality contrast (HENETIX^®^; Guerbet, Rio de Janeiro, Brazil) medium was used; the amount required was calculated based on the patient's weight. In the standard protocol, the patient was first positioned with the head and neck in a neutral (anatomic) position. The acquisition protocol consisted of a bolus test phase (axial sequential image acquisition in the basilar artery region, with the objective of monitoring contrast medium dynamics and determining the best arterial peak enhancement) and an angiographic phase. Anatomical coverage of the acquisition was from the aortic arch to the circle of Willis. The second half of the contrast medium bolus was administered with the head and neck rotated to the side that triggered the symptoms (left side in the present case) to acquire another arterial phase with technical parameters similar to the neutral position acquisition.

All detailed technical parameters for acquiring dynamic CTA are provided in table 1.

**Table 1:** Dynamic computed tomography angiography acquisition protocol for bow hunter's evaluation - technical parameters

	Detectors / Collimation / Rot time	Slice thickness (mm)	Kv	Tube current (mA)	FOV (mm)	Range (mm)	Pitch	Filter	Dose modulation	Contrast injection
Test Bolus	4/0.5/0.5	2.0/~	80	100	240	~	~	CTA Neck (FC 43)	~	4–5mL/s Volume: 15mL
Neutral arterial phase	80/0.5/0.35	1.0/0.8	120	Dose modulation	240	250	0.9	CTA Neck (FC 43)	Sure Exposure AIDR 3D Quality (SD 10, mA 200–500)	4–5mL/s Volume: 1mL/Kg
Head and neck rotation arterial phase	80/0.5/0.35	1.0/0.8	120	Dose modulation	240	250	0.9	CTA Neck (FC 43)	Sure Exposure AIDR 3D Quality (SD 10, mA 200–500)	4–5mL/s Volume: 1mL/Kg

Since the dynamic CTA protocol for BHS evaluation requires the patient to be placed in a position that reproduces symptoms, if symptoms occur during the study, the assisting team should carefully return the head and neck to a neutral position to avoid complications. A drawback of this technique is that it involves more radiation exposure and a longer execution time in comparison with conventional CTA; however, both are under reasonable limits, making the benefits surpass the risks.

To generate a 3D-printed model, two software programs dedicated to the analysis and segmentation of medical images for research were used (Mimics and 3-Matic; Materialise NV, Leuven, Belgium). The tomographic images were volumetric and isotropic, with a thickness of 1mm and an increment of 0.8mm, allowing the segmentation of bones and arterial vessels. With the segmentation ready, a file in the stereolithography format was sent to a 3D printer with fused deposition modeling technology. The material selected for printing was acrylonitrile butadiene styrene, which is the plastic polymer most commonly used in 3D printing. The color chosen was natural one for the bone, and red for the arteries.

This study was approved by the Research Ethics Committee of *Hospital Israelita Albert Einstein* (CAAE: 79086324.9.0000.0071; #6.842.007) and was conducted in accordance with the ethical standards of the institutional research committee and the 1964 Helsinki Declaration and its later amendments or comparable ethical standards. Moreover, patient informed consent was obtained.

## RESULTS

**Figure 1:**
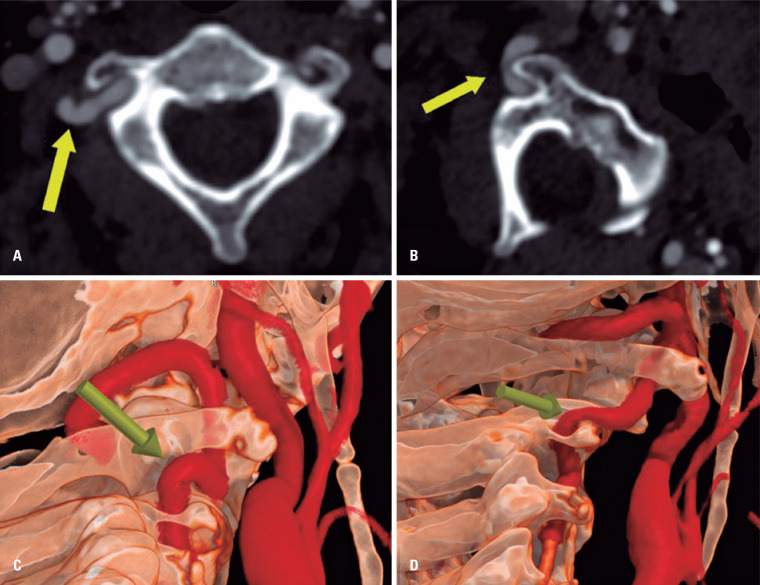
(A) Computed tomography angiography image of axial neutral arterial phase demonstrates a dominant right vertebral artery, with a marked tortuosity in the transition of the V2 to V3 segment, without significant stenosis (arrow in A and C); (B) After left head and neck rotation (position which patient referred symptoms), severe stenosis can be observed in the V2/V3 transition due to compression by the superolateral aspect of the right transverse process of C2; (C) 3D volume rendering image shows the anatomic position without stenosis and (D) after head and neck rotation showing significant stenosis (arrow in B and D)

Axial source images, multiplanar reformatted images, maximum intensity projections, curved reformatted images, and 3D volume-rendered images were used for image interpretation. A summary of the main findings is shown in figure 1, which provides a clear depiction of the regional anatomy, focal severe stenosis of the V2/V3 segments of the right VA present only in the left head/neck rotation position, and a bony structure related to the compression of the right VA segment.

**Figure 2:**
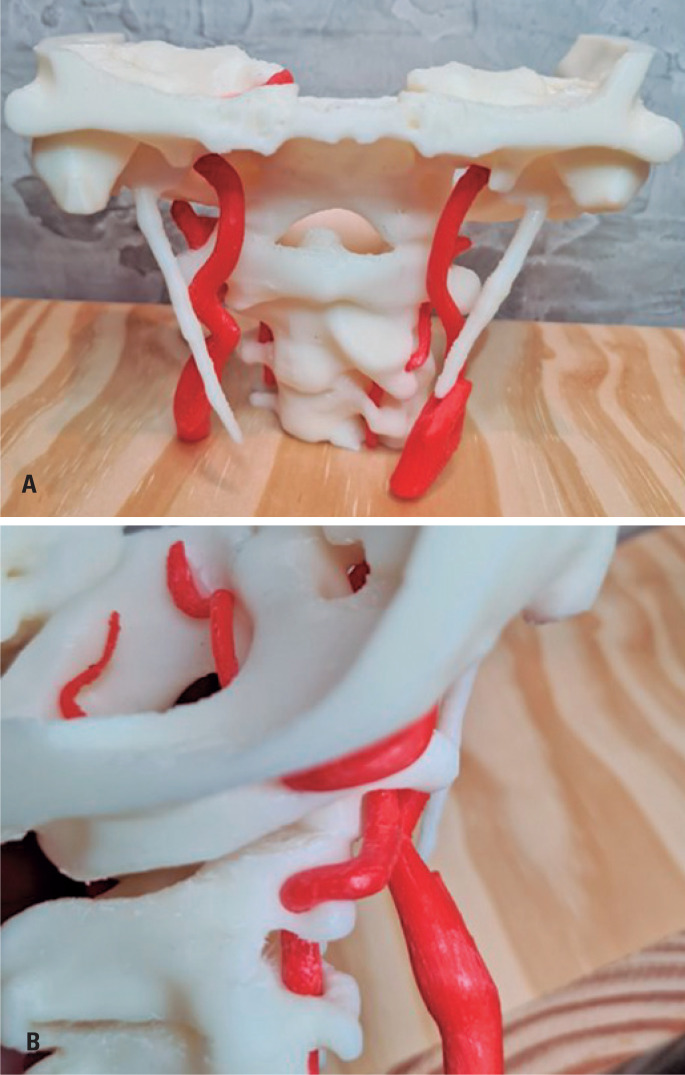
Anterior coronal view (A) and left lateral view (B) of the 3D-printed model of the left rotated head and neck Computed tomography acquisition showing the relation between the main cervical arteries with regional bone structures. Right lateral view (B) focuses on showing the relation between the left C2 transverse process and the V2/V3 transition of the right vertebral artery

Dynamic CTA radiologic reports included vascular evaluation (focusing on the observation of a dominant VA artery, circle of Willis anatomy, and detection of stenosis caused or worsened by head and neck rotation) and bone evaluation (bone, ligament, or membranous structures related to vascular compression during head and neck rotation). Good quality images obtained via dynamic CTA acquisition allowed segmentation and generation of a 3D-printed model, with the objective of better depicting the findings and helping the surgical team program the treatment procedure (Figure 2).

## DISCUSSION

The present study contributes to existing literature by providing a detailed technical description of a dynamic CTA study for the noninvasive evaluation of BHS, which consisted of image acquisition in a neutral position and in a neck rotation position, similar to that used to investigate thoracic outlet syndrome. The acquisition protocol is illustrated by the figures previously presented for a confirmed BHS case. Additionally, the value of this technical note is reinforced by the generation of a 3D-printed model generated to aid in treatment planning.

Dynamic DSA, which is considered the gold standard for the diagnosis of BHS, and dynamic CTA might have complementary role in the disease. In a patient suspected of having BHS, we suggest initiate the investigation of the disease with Doppler sonography and/or Dynamic CTA/MRA. Digital subtraction angiography still has an important role in diagnosis of complex cases and in endovascular treatment of the disease. 3D printed models are not widely available and we suggest considering its utilization after multidisciplinary team discussion.

## CONCLUSION

In conclusion, dynamic computed tomography angiography is a feasible noninvasive method that provides alternatives for the diagnosis and evaluation of bow Hunter's syndrome, and the present study provides technical parameters to reproduce acquisition. The images obtained using the proposed protocol are available, and may serve as reference standards. Finally, computed tomography angiography also allows the generation of 3D printed models, which may help in surgical procedure planning.
